# Effect of Low Nitrogen on Photosynthesis, Physiology, and Mineral Element Responses of Self-Grafted and Grafted Citrus Seedlings

**DOI:** 10.3390/plants15121841

**Published:** 2026-06-14

**Authors:** Ling Liao, Ziyi Huang, Wenjing Xia, Feiyi Li, Yunjie Li, Xinya Zhou, Mingfei Zhang, Siya He, Xun Wang

**Affiliations:** College of Horticulture, Sichuan Agricultural University, Chengdu 611130, China

**Keywords:** citrus rootstock, grafting, nitrogen deficiency, photosynthesis, mineral elements

## Abstract

Grafting is a widely used technique to improve stress tolerance in horticultural plants. However, little is known about how grafting affects citrus growth under low-nitrogen (N) stress. To investigate the responses of different grafting combinations to low N availability, we examined root morphology, photosynthesis, chlorophyll fluorescence and semi-quantitative mineral profiles in grafted and ungrafted citrus plants subjected to two N levels (10 and 0.15 mM NO_3_^−^ -N) under potted conditions. Analyses were performed on roots and leaves of six plant combinations: ungrafted Trifoliate orange (*Poncitrus trifoliata* L. Raf., Pt) and red tangerine (*Citrus reticulata* Blanco, Cr); self-grafted combinations (Pt/Pt and Cr/Cr); and reciprocal heterografts (Pt/Cr and Cr/Pt). Under low-N stress, plant height decreased by 12.3–36.8%, stem diameter by 2.9–31.8%, leaf area by 18.2–26.3%, and SPAD by 11.6–24.5% across the six combinations, with the Cr/Cr combination showing the largest reductions in all parameters. The highest net photosynthetic rate (*P*_n_), intercellular CO_2_ concentration (C_i_), stomatal conductance (Gs), electron transport rate (ETR), maximum quantum efficiency of PSII (Fv/Fm) and effective quantum efficiency of PSII (Fv’/Fm’) were observed in the Pt and Pt/Pt plants. Low-N stress reduced chloroplastid pigment contents and limited photosynthetic rates. Under 10 mM N treatment, the Fv/Fm values of Pt, Cr, Pt/Pt, and Cr/Pt were approximately 0.82, whereas those of Pt/Cr and Cr/Cr were below 0.82, suggesting lower maximal PSII efficiency in combinations with Cr rootstock. Regarding mineral elements, under low-N stress, the relative levels of P, K, Ca, Mg, and Fe in leaf and root sap increased, while those of N, Cu, Zn, B, and Mn decreased. Overall, combinations with Pt rootstock (Pt/Pt and Cr/Pt) showed better growth and photosynthetic performance, and more stable mineral profiles under low-N stress than combinations with Cr rootstock (Cr/Cr and Pt/Cr). These findings provide a physiological basis for understanding rootstock-specific responses to low-N stress under controlled conditions.

## 1. Introduction

Citrus is one of the most extensively cultivated fruit crops globally and is adaptable to various climate and soil types [1]. China ranks as the top citrus producer worldwide, accounting for 32% of the global cultivation area and 28% of global output (FAOSTAT, 2024) [2]. In China, citrus cultivation is concentrated in the southern tropical and subtropical zones, where the typical red soils are inherently low in fertility [3]. Nitrogen (N) is an indispensable mineral element for plants. It forms a key constituent of proteins, nucleic acids, phospholipids, chlorophyll, hormones, vitamins and alkaloids, and is involved throughout the entire plant life cycle [4]. Nitrogen deficiency frequently limits plant growth in both natural ecosystems and agricultural settings, as soil erosion exacerbates nutrient depletion and leads to a decline in soil N content [5]. Under low-N stress, plants typically exhibit reduced photosynthetic rates, earlier leaf senescence, and slower biomass accumulation, ultimately leading to irreversible damage [6]. Uneven N distribution within the soil can affect the morphological development of plant roots [7]. In *Arabidopsis*, N application increases resource partitioning to leaves and stems at the expense of roots and fruits [8]. For fruit trees, N application may enhance production output and improve fruit quality attributes [9]. It has been demonstrated that either insufficient or excessive N fertilization can lead to poor growth and yield instability in citrus trees [10]. Motivated by economic gains, farmers have adopted fertilization as the main approach to increase citrus production in China [11]. A nationwide survey reported that nitrogen fertilizer is applied to citrus orchards at an average rate as high as 500 kg ha^−1^ [12]. The over-application of N and other nutrients in China’s low-yielding citrus orchards far exceeds tree requirements, resulting in poor fertilizer use efficiency [13], severe soil acidification, imbalanced soil nutrients [3] and environmental risks [11].

Grafting is recognized as a viable method for improving nitrogen nutrition in plants [14]. It is an effective strategy to enhance abiotic stress tolerance by optimizing root–scion signaling, nutrient uptake, photosynthetic efficiency, and nitrogen metabolism in horticultural crops [15]. Citrus has been cultivated commercially using grafted plants for centuries [16]. Canopy structure and root system distribution are influenced by the combination of scion and rootstock varieties [17]. Roots store N and C reserves, absorb and assimilate nutrients from the soil, and deliver them to the aerial parts via the xylem; therefore, using suitable and compatible rootstocks can improve water and nutrient acquisition and nutrient use efficiency [18]. Rootstocks affect ion acquisition in grafted plants. In kinnow mandarin, grafting onto nine rootstocks increased leaf N, P, and K by 4–18%, 11–77%, and 3–43%, respectively, and boosted per-plant fruit count to 0.12–5.63 times that of a low-performing rootstock [19]. Total nitrogen accumulation and utilization efficiency also vary significantly among citrus rootstocks. For instance, rough lemon (*Citrus jambhiri* Lush.) accumulated a total nitrogen value of 22.1 mg/g DW, whereas Cleopatra mandarin (*Citrus reshni hort.* ex Tanaka) accumulated only 6.1 mg/g DW [20]. Scions can also affect rootstock growth and development [21]. Several studies have investigated how nutrients, metabolites, and signaling molecules are translocated from shoots to roots via the phloem [22,23]. Consequently, the levels of photosynthates, metabolites, and phytohormones in shoots may strongly affect root growth and development in grafted plants.

The use of rootstocks in fruit plants affects tree vigor and size, precocity, fruit quality and taste, harvestable yield, pest resistance, and tolerance to edaphic and environmental conditions by invigorating the scions and increasing nutrient uptake, transport, and utilization efficiency [24]. Rootstock–scion interactions have demonstrated that rootstock often has a greater influence than the scion on tree weight and growth rate [25]. However, poor interactions between rootstock and the scion may also become a barrier to water and mineral nutrient translocation, favoring the formation of calluses that may affect some physiological and biochemical processes in the plant [26]. Trifoliate orange (*Poncitrus trifoliata* L. Raf., Pt) and red tangerine (*Citrus reticulata* Blanco, Cr) are important citrus rootstocks used in the southwest region of China [27]. Previous studies have primarily focused on the effects of different rootstock–scion combinations on scion growth and fruit quality [28], while research on how different rootstocks influence nitrogen utilization and distribution in citrus plants remains limited [29]. Nitrogen deficiency restricts chlorophyll synthesis, impairs PSII function, reduces photosynthetic electron transport, and disrupts mineral nutrient balance. The rootstock genotype is known to affect tissue nitrogen concentration and mineral element profiles under low-N stress. However, the physiological basis by which citrus rootstocks mediate photosynthesis, nitrogen metabolism, and mineral element distribution under low-N stress remains unclear. This study aimed to investigate how different grafting combinations affect growth, photosynthetic performance, and relative mineral element levels in grafted citrus seedlings under low-N stress. We tested two hypotheses: (1) under low-N stress, combinations with Pt rootstock (Pt/Pt and Cr/Pt) show better growth and photosynthetic performance than those with Cr rootstock (Cr/Cr and Pt/Cr); (2) low-N stress alters relative mineral element levels in a grafting-combination-dependent manner.

## 2. Materials and Methods

### 2.1. Plant Materials, Grafting, and Experimental Design

The experiment was conducted at the campus of Sichuan Agricultural University in Chengdu, China (annual average temperature < 20 °C; annual rainfall 760 mm). One-year-old seedlings of Trifoliate orange (*Poncitrus trifoliata* L. Raf., Pt) and red tangerine (*Citrus reticulata* Blanco, Cr) were used as both rootstocks and scions.

Grafting procedure: Grafting was performed outdoors in March using the single-bud cut grafting method. For each grafting combination, rootstock seedlings were cut horizontally at 10 cm above the root collar. A 2 cm vertical or slightly slanted cut was made at the center of the cut surface. Scions (single buds with a 1–2 cm stem piece) were collected from 1-year-old seedlings of the same species and trimmed into a wedge shape at the base to match the rootstock cut. The wedge was inserted into the rootstock cut, and the graft union was wrapped tightly with grafting film. The natural outdoor conditions during the grafting period (spring in Chengdu) were suitable for graft healing. The graft success rate was >95% for all combinations. Graft survival was assessed 20 days after grafting based on scion bud freshness and graft union healing.

Growing conditions: After grafting, plants were transplanted into plastic pots (30 cm × 20 cm × 26 cm) filled with a sterile substrate of vermiculite and perlite (volume ratio 3:1). Plants were irrigated with half-strength Hoagland nutrient solution (pH 6.0) every 3 days, with 200 mL per pot each time.

Treatments and plant selection: Six grafting combinations were established: ungrafted Pt (Pt), ungrafted Cr (Cr), Pt grafted onto Pt (Pt/Pt), Cr grafted onto Cr (Cr/Cr), Pt grafted onto Cr (Pt/Cr), and Cr grafted onto Pt (Cr/Pt). When most plants in each combination had grown 20 fully unfolded leaves, 18 plants of uniform growth potential were selected per combination. These 18 plants were divided into three groups of six plants each, and all measurements were performed with three biological replicates, with each replicate consisting of two plants. Group 1 (six plants) was used for gas exchange, chlorophyll fluorescence, light-response and CO_2_ response curves, and chlorophyll content. Group 2 (six plants) was used for plant growth parameters (height, stem diameter, leaf area) and root morphology. Group 3 (six plants) was used for mineral element analysis (leaves and roots from the same plants). For each biological replicate (two plants), the following sampling procedures were applied: for gas exchange, chlorophyll fluorescence, and light/CO_2_ response curves, one fully expanded leaf was sampled from one of the two plants per replicate; the second plant was not used for these measurements. The plant used for these measurements was selected randomly from the two plants in each replicate. For chlorophyll content and mineral element analysis, leaf and root tissues from both plants in the replicate were pooled before extraction or sap preparation, and measurements were performed on the pooled sample. For root morphology, roots from both plants were scanned separately and the values averaged per replicate.

Nitrogen treatment: Two nitrogen levels were applied: 10 mM NO_3_^−^-N (control) and 0.15 mM NO_3_^−^-N (low N). The control solution contained 3 mM Ca(NO_3_)_2_ and 4 mM KNO_3_; the low-N solution contained 0.15 mM KNO_3_, 3 mM CaCl_2_, and 3.85 mM KCl to maintain K^+^ and Ca^2+^ levels. Both solutions shared a common base: 0.5 mM KH_2_PO_4_, 1.5 mM K_2_SO_4_, 1 mM MgSO_4_·7H_2_O, 0.05 mM FeSO_4_, 0.05 mM EDTA-Na_2_, plus micronutrients (H_3_BO_3_, MnCl_2_, ZnSO_4_, CuSO_4_, H_2_MoO_4_). Plants were cultured for 8 weeks, with the solution aerated for 2 h each day and replaced once per week. The pH was adjusted to 6.0 with 0.1 M KOH. The experiment had a completely randomized design with two nitrogen levels × six grafting combinations.

### 2.2. Plant Growth and Root Morphology

Plant height was measured using a tape measure. Stem diameter was measured at 1 cm above the graft union (or at the corresponding position in ungrafted plants) using an electronic vernier caliper. Leaf area and leaf SPAD (relative chlorophyll content) were measured using an LI-3100C area meter (Li-Cor Inc., Lincoln, NE, USA)and a SPAD meter (SPAD-502; Konica Minolta, Tokyo, Japan), respectively. Root scanning was performed using an Epson Expression 10000 XL scanner (Seiko Epson Corp., Suwa, Nagano, Japan). Total root length, root surface area, average root diameter, root volume, root tip number, and root fork number were measured using WinRHIZO 2009 (Regent Instruments Inc., Quebec City, QC, Canada).

Root activity was determined using the 2,3,5-triphenyltetrazolium chloride (TTC) reduction method [30]. Fresh root tips (0.2 g) were incubated in 10 mL of a reaction mixture (0.4% TTC in 0.1 M phosphate buffer, pH 7.0, 1:1, *v*/*v*) at 37 °C for 2 h in the dark. After removing the solution, the reduced triphenylformazan (TTF) was extracted with 10 mL of 95% ethanol at 80 °C for 30 min. The absorbance of the extract was measured at 485 nm (Model UV-1600, Shimadzu Corporation, Kyoto, Japan). A standard curve was prepared using TTF standards. Root activity was expressed as µg·g^−1^·h^−1^ fresh weight (FW). Three biological replicates (each with pooled roots from two plants) were measured.

### 2.3. Mineral Element Analysis

Fresh leaf and root samples were washed with deionized water and blotted dry. The samples were cut into 1–2 mm fragments, mixed, and ground in a mortar until cell sap was released. Two drops of the sap were transferred into a 10 mL graduated cylinder and diluted with deionized water to 10 mL (100-fold dilution). Relative levels of available N (NO_3_^−^-N), P, K, S, Fe, Mn, B, Zn, Cu, Ca, and Mg were determined using a YT-ZY20 plant nutrition tester (Shandong Yuntang Intelligent Technology Co., Ltd., Weifang, Shandong, China) following the manufacturer’s protocols. For each element, a 2 mL aliquot of the diluted sap was reacted with the corresponding reagent kit, and the absorbance (or titration endpoint for Ca and Mg) was measured according to the instrument manual. The instrument was calibrated with the standard solutions provided with each kit (external standard method); a reagent blank was included in each batch. The instrument operates on the Lambert–Beer law with a measurement range of 0.001–9999 and a repeatability error of ≤0.05%. All measurements were performed in triplicate per pooled sample. This method provides semi-quantitative relative comparisons of mineral element levels in fresh sap, rather than absolute tissue ionomic concentrations. The instrument outputs values in mg·kg^−1^ fresh weight as a relative scale. We did not validate these measurements against ICP-OES, and the results should be interpreted as comparative profiles across treatments rather than absolute mineral contents. For each biological replicate, tissues from two plants were pooled before sap extraction.

### 2.4. Gas Exchange, Chlorophyll Fluorescence and Chlorophyll Content

All measurements were taken with a Li-Cor 6800 portable photosynthesis system (Li-Cor Inc., Lincoln, NE, USA). Light-saturated photosynthesis was measured from 09:00 to 11:30 h under leaf chamber conditions: PPFD of 1000 µmol·m^−2^·s^−1^, CO_2_ of 400 ± 2 µmol·mol^−1^, and gas flow rate of 500 µmol·s^−1^. Data were recorded in steady state (~15 min). One leaf per biological replicate (from one of the two plants) was measured.

Light-response curves: Leaves were acclimated at PPFD of 1200 µmol·m^−2^·s^−1^ for 10–15 min, then PPFD was decreased stepwise to 2000; 1800; 1500; 1200; 1000; 800; 500; 200; 150; 100; 50; and 0 µmol·m^−2^·s^−1^. CO_2_ response curves were measured after the light-response curve under the same PPFD (1200 µmol·m^−2^·s^−1^), with CO_2_ stepped as follows: 400, 300, 200, 100, 50, 0, 400, 400, 600, 800, 1000, 1200, 1500 and 2000 µmol·mol^−1^ (2~3 min per step). Light-response curves were fitted using the Ye model [31], and CO_2_ response curves were fitted using the modified rectangular hyperbola models [32]. All fits had R^2^ > 0.95. Measurements were performed on three leaves per treatment (one leaf per biological replicate).

Chlorophyll fluorescence was measured on the same leaves. For dark-adapted Fv/Fm, leaves were dark-adapted for 30 min, and Fv/Fm was calculated as (Fm-Fo)/Fm. For light-adapted measurements, steady-state fluorescence (Fs), and maximum fluorescence (Fm’) were recorded at PPFD of 1000 µmol·m^−2^·s^−1^ and CO_2_ of 400 µmol·mol^−1^; Fm’ was measured with a 0.8 s saturating light pulse. Effective quantum efficiency of PSII in the light (Fv’/Fm’) was calculated as (Fm’−Fs)/Fm’.

Chlorophyll content: Leaves were chopped into 1 mm segments. Subsamples (0.1 g) were placed in test tubes with 8 mL of 80% acetone, sealed, and extracted in darkness for 24 h until completely bleached. Absorbance of *chl a* and *chl b* was measured at 663 nm and 645 nm (UV-1600, Shimadzu, Japan). Pigment concentrations were calculated with the Arnon formula [33]. Three biological replicates (each with pooled leaf tissue from two plants) were measured.

### 2.5. Statistical Analysis

Data were analyzed by two-way ANOVA for a 2 × 6 factorial design (N level × graft combination). Main effects of N, graft combination, and their interaction (N × G) were determined. Means were compared by Tukey’s HSD test at *p* < 0.05. For gas exchange, chlorophyll fluorescence, and light/CO_2_ response parameters, the effective experimental unit was one leaf from one plant per biological replicate (*n* = 3). For all other measurements, the effective sample size was also *n* = 3, with pooling or averaging of tissues from the two plants as described in Section 2.1. All analyses were performed in SPSS 22.0 (IBM Corp., Armonk, NY, USA).

## 3. Results

### 3.1. Differences in Phenotypes Among the Six Citrus Combinations

**Figure 1:**
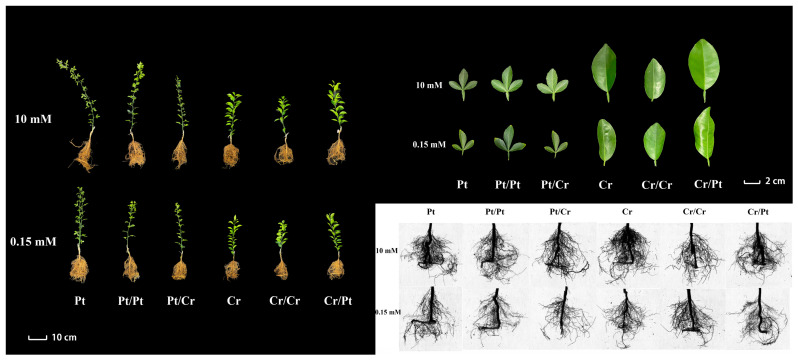
Phenotypes of leaves, roots, and whole plants of six citrus combinations under two nitrogen regimes. The six citrus combinations are: Ungrafted Trifoliate orange (*Poncitrus trifoliata* L. Raf., Pt), ungrafted red tangerine (*Citrus reticulata* Blanco, Cr); self-grafted combinations (Pt/Pt and Cr/Cr); reciprocal heterografts (Pt/Cr and Cr/Pt). Scale bars = 10 cm (whole plant) and 2 cm (leaf). Root images were acquired using an Epson Expression 10000 XL scanner at 300 dpi; root morphological parameters were quantified using WinRHIZO 2009 software (see Table 2 for quantitative data).

Plant height, stem diameter, SPAD (relative chlorophyll content), and leaf area for the six grafting combinations (ungrafted Pt, ungrafted Cr, Pt/Pt, Cr/Cr, Pt/Cr, and Cr/Pt) are presented in Table 1. Grafting affected the development and growth of the scions, rootstocks, and whole plant, as evidenced by morphological differences. Under both 10 mM N and 0.15 mM N treatments, plant height, stem diameter, and SPAD were significantly higher in Pt plants than in Cr plants, whereas leaf area was significantly larger in Cr plants than in Pt plants (Figure 1).

Under 10 mM N treatment, compared with Pt, plant height decreased by 3.92% in Pt/Pt and by 24.14% in Pt/Cr; compared with Cr, plant height decreased by 21.55% in Cr/Cr but increased by 29.98% in Pt/Cr. Stem diameter, leaf area and SPAD were also affected by grafting to varying degrees (Table 1). Low N reduced plant height, stem diameter, leaf area and the SPAD in all combinations, with combinations using Cr rootstock (Cr/Cr and Pt/Cr) showing the largest reductions. Compared with the 10 mM N treatment, under 0.15 mM N, plant height decreased by 12.3%~36.8%, stem diameter by 2.9%~31.8%, leaf area by 8.2%~26.3%, and SPAD by 11.6%~24.5% across the six combinations.

### 3.2. Root Morphology

Total root length, surface area, root tip number and root activity were significantly higher in Pt plants than in Cr plants under both N treatments, whereas average root diameter was larger in Cr plants than in Pt plants (Table 2). Root volume was significantly greater in Pt than in Cr under 10 mM N, but the opposite pattern was observed under 0.15 mM N. Under 10 mM N, compared with Pt, most root morphology indicators and root activity were significantly reduced in Pt/Pt and Pt/Cr, except for root tip number. Compared with Cr, total root length, surface area, and root tip number increased in Cr/Cr and Cr/Pt, whereas root diameter, root volume, and root activity showed variable patterns. Low-N stress reduced all root morphology indicators and root activity across all six combinations, with the largest reductions observed in combinations using Cr rootstock (Cr/Cr and Pt/Cr).

### 3.3. Chlorophyll Content

**Figure 2:**
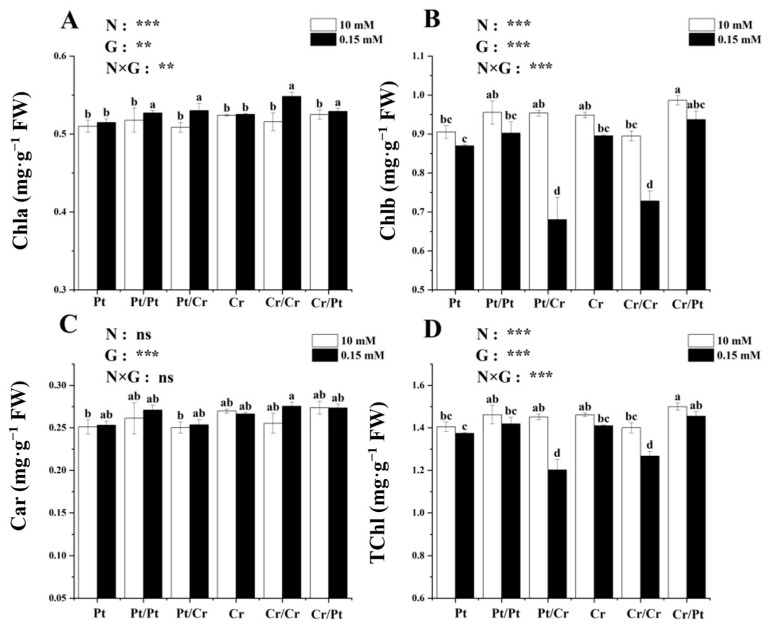
Concentrations of (**A**) Chlorophyll a, (**B**) Chlorophyll b, (**C**) total carotenoid, and (**D**) total chlorophyll in leaves of the six citrus combinations under two N regimes. The six citrus combinations are: Ungrafted Trifoliate orange (*Poncitrus trifoliata* L. Raf., Pt), ungrafted red tangerine (*Citrus reticulata* Blanco, Cr), Pt grafted onto Pt (Pt/Pt), Cr grafted onto Cr (Cr/Cr), Pt grafted onto Cr (Pt/Cr), Cr grafted onto Pt (Cr/Pt). ** *p* < 0.01; *** *p* < 0.001; ns, not significant. *n* = 3 biological replicates, with leaf tissue from two plants pooled per replicate; different letters indicate significant differences determined by Tukey test at *p* < 0.05.

No significant differences in *chl a*, *chl b*, total chlorophyll or carotenoid contents were observed between Pt and Cr leaves under either N treatments. As shown in Figure 2, under 10 mM N, there were no significant differences among the six combinations in *chl a*, *chl b*, total chlorophyll or carotenoids, except that *chl b* and total chlorophyll were significantly higher in Cr/Pt than in Cr/Cr and Pt. Under 0.15 mM N, *chl a* content increased significantly in Pt/Pt, Cr/Cr, Pt/Cr, and Cr/Pt, whereas *chl b* content decreased significantly in Cr. *chl b* content was 32.3% higher in Pt/Pt than in Pt/Cr, and 29.2% higher in Cr/Pt than in Cr/Cr; total chlorophyll content was 17.5% higher in Pt/Pt than in Pt/Cr, and 15.1% higher in Cr/Pt than in Cr/Cr.

### 3.4. Leaf Gas Exchange and Chlorophyll Fluorescence

As shown in Table 3, the net photosynthetic rate (*P*_n_), intercellular CO_2_ concentration (C_i_), stomatal conductance (Gs), electron transport rate (ETR), maximum quantum efficiency of PSII (Fv/Fm), and effective quantum efficiency of PSII (Fv’/Fm’) were all significantly higher in Pt plants than in Cr plants under both N treatments. The ETR decreased in grafted seedlings compared with their respective ungrafted controls. The highest ETR values were observed in Pt leaves, followed by Pt/Pt leaves, whereas Cr/Cr leaves exhibited the lowest values under both N treatments. Under 10 mM N treatment, Fv/Fm values for Pt, Cr, Pt/Pt, and Cr/Pt were approximately 0.82, whereas those for Pt/Cr and Cr/Cr were below 0.82, suggesting that combinations with Cr as rootstock may be more susceptible to reduced PSII efficiency.

Grafting reduced photosynthetic performance. Compared with Pt, *P*_n_ decreased by 12.93% in Pt/Pt and by 24.40% in Pt/Cr; compared with Cr, *P*_n_ decreased by 16.18% in Cr/Cr and by 21.89% in Cr/Pt. Low-N stress reduced all gas exchange and chlorophyll fluorescence parameters across the six combinations, with the largest reductions in combinations using Cr rootstock. Under 0.15 mM N, *P*_n_, Ci, Gs and the ETR were 34.8%, 33.4%, 17.9% and 27.5% higher, respectively, in Pt/Pt than in Pt/Cr. Similarly, *P*_n_, Ci, Gs, ETR and Fv‘/Fm’ were significantly higher in Cr/Pt than in Cr/Cr by 18.9%, 14.3%, 28.2%, 25% and 18.7%, respectively.

### 3.5. Photosynthetic Light-Response Curves

**Figure 3:**
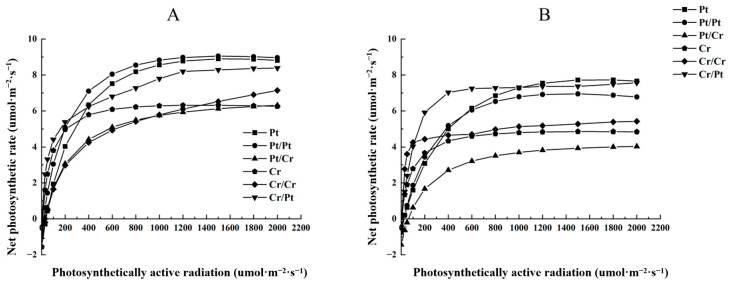
Photosynthetic light-response curves of six citrus combinations under (**A**) 10 mM N and (**B**) 0.15 mM N. The six citrus combinations are: Ungrafted Trifoliate orange (*Poncitrus trifoliata* L. Raf., Pt), ungrafted red tangerine (*Citrus reticulata* Blanco, Cr), Pt grafted onto Pt (Pt/Pt), Cr grafted onto Cr (Cr/Cr), Pt grafted onto Cr (Pt/Cr), Cr grafted onto Pt (Cr/Pt). *n* = 3 biological replicates, with one leaf measured from one of the two plants per replicate.

The response of *P*_n_ to photosynthetic photon flux density (PPFD) for the six citrus combinations is shown in Figure 3. Under both N treatments, Pt plants exhibit significantly higher maximum photosynthetic rates at light saturation (LS*P*_n_), the light compensation point (LCP), and apparent quantum efficiency (AQY) than Cr plants. Under 10 mM N, the light saturation point (LSP) ranged from 933.86 to 1705.81 µmol m^−2^·s^−1^ across the six combinations. Compared with Pt, LS*P*_n_ remained almost unchanged in Pt/Pt but decreased significantly in Pt/Cr; LCP decreased in Pt/Pt but increased in Pt/Cr. Compared with Cr, LS*P*_n_ remained almost unchanged in Cr/Cr but increased significantly in Cr/Pt. Low-N stress reduced LS*P*_n_, LSP, LCP and AQY in all combinations (Table 4). In the light-response parameters, Pt/Pt and Cr/Pt generally performed better than Pt/Cr and Cr/Cr, respectively. For example, compared with Pt, LS*P*_n_ decreased by 11.5% in Pt/Pt and by 45.4% in Pt/Cr; compared with Cr, LS*P*_n_ increased by 10.9% in Cr/Cr and by 49.0% in Cr/Pt.

### 3.6. Photosynthetic CO_2_ Response Curves

**Figure 4:**
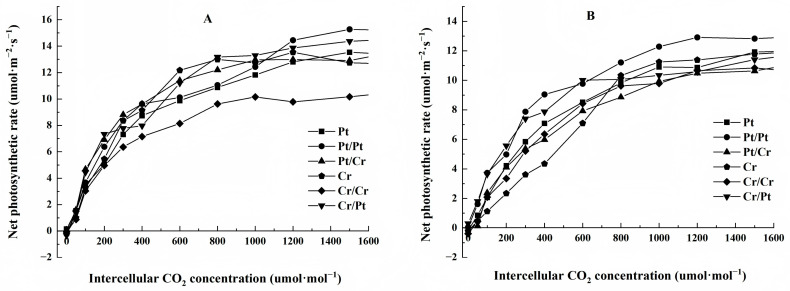
Photosynthetic CO_2_ response curves of six citrus combinations under (**A**) 10 mM N and (**B**) 0.15 mM N. The six citrus combinations are: Ungrafted Trifoliate orange (*Poncitrus trifoliata* L. Raf., Pt), ungrafted red tangerine (*Citrus reticulata* Blanco, Cr), Pt grafted onto Pt (Pt/Pt), Cr grafted onto Cr (Cr/Cr), Pt grafted onto Cr (Pt/Cr), Cr grafted onto Pt (Cr/Pt). *n* = 3 biological replicates, with one leaf measured from one of the two plants per replicate.

The response of *P*_n_ to intercellular CO_2_ concentration (*C*_i_) is shown in Figure 4. Under both N treatments, there were no significant differences in the maximum photosynthetic rate at CO_2_ saturation (CS*P*_n_) between Pt and Cr plants. Under 10 mM N, the maximum carboxylation rate limited by Rubisco (Vcmax), CO_2_ saturation point (CSP), and CO_2_ compensation point (CCP) were significantly higher in Pt than in Cr plants. CS*P*_n_ ranged from 10.18 to 16.57 μmol·m^−2^·s^−1^ across the six combinations. Compared with Pt, CS*P*_n_ increased by 23.3% in Pt/Pt but decreased by 10.4% in Pt/Cr; compared with Cr, CS*P*_n_ decreased by 17.2% in Cr/Cr but increased by 11.5% in Cr/Pt (Table 5). Low-N stress reduced CS*P*_n_, Vcmax, and CSP in all combinations, while CCP increased. Compared with Pt, CS*P*_n_ increased by 29.7% in Pt/Pt and by 13.0% in Pt/Cr; compared with Cr, CS*P*_n_ decreased by 18.0% in Cr/Cr and 9.3% in Cr/Pt (Table 5).

### 3.7. Relative Levels of Major Elements

**Figure 5:**
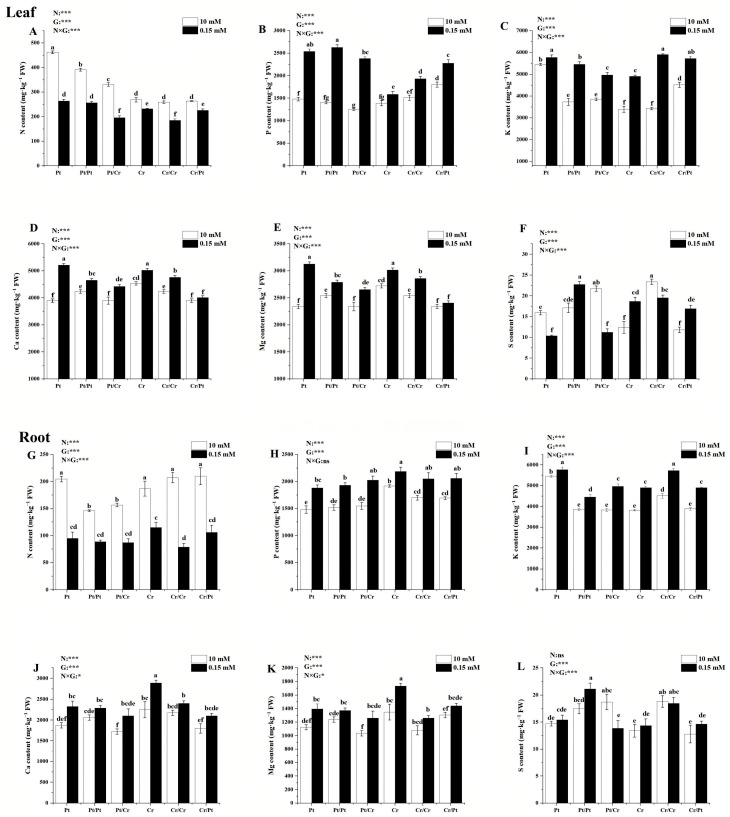
Relative levels of major elements (N, P, K, Ca, Mg, S) in leaves and root of six citrus combinations under two N regimes. (**A**) Leaf N levels; (**B**) Leaf P levels; (**C**) Leaf K levels; (**D**) Leaf Ca levels; (**E**) Leaf Mg levels; (**F**) Leaf S levels; (**G**) Root N levels; (**H**) Root P levels; (**I**) Root K levels; (**J**) Root Ca levels; (**K**) Root Mg levels; (**L**) Root S levels. The six citrus combinations are: Ungrafted Trifoliate orange (*Poncitrus trifoliata* L. Raf., Pt), ungrafted red tangerine (*Citrus reticulata* Blanco, Cr), Pt grafted onto Pt (Pt/Pt), Cr grafted onto Cr (Cr/Cr), Pt grafted onto Cr (Pt/Cr), Cr grafted onto Pt (Cr/Pt). Y-axis values are instrument outputs from diluted fresh sap (YT-ZY20 tester) and represent semi-quantitative relative comparisons; they were not validated by ICP-OES. Statistical significance: * *p* < 0.05; *** *p* < 0.001; ns, not significant. Different letters indicate significant differences determined by Tukey test at *p* < 0.05. *n* = 3 biological replicates, with leaf or root tissues from two plants pooled per replicate before sap extraction.

Except for S, the relative levels of N, P, K, Ca, and Mg in leaves were significantly affected by N treatment, graft combination, and their interaction (N × G). Under 10 mM N, relative leaf levels of N, K and S were significantly higher in Pt than in Cr (by 71.7%, 37.8% and 22.3%, respectively), whereas Ca and Mg levels were significantly lower in Pt than in Cr (by 14.1% and 13.8%, respectively). No significant differences in P levels were observed between Pt and Cr leaves. Among grafted combinations, relative levels of N, P, Ca, and Mg were higher in Pt/Pt leaves than in Pt/Cr leaves, whereas K and S levels were lower in Pt/Pt than in Pt/Cr. In Cr/Pt leaves, relative levels of N, P, and K were higher than in Cr/Cr leaves, whereas Ca, Mg, and S levels were lower. Similar patterns were observed in roots (Figure 5).

When N concentration decreased from 10 mM to 0.15 mM, relative N levels in leaves and roots decreased significantly, by 14.1–43.0% and 38.7–62.3%, respectively, whereas relative levels of P, K, Ca, and Mg increased. Sulfur responses varied by grafting combination: in leaves, S levels decreased in Pt, Pt/Cr, and Cr/Cr but increased in Cr, Pt/Pt, and Cr/Pt under low N; in roots, S levels decreased in Pt/Cr and Cr/Cr but increased in Pt, Cr, Pt/Pt, and Cr/Pt.

### 3.8. Relative Levels of Trace Elements

**Figure 6:**
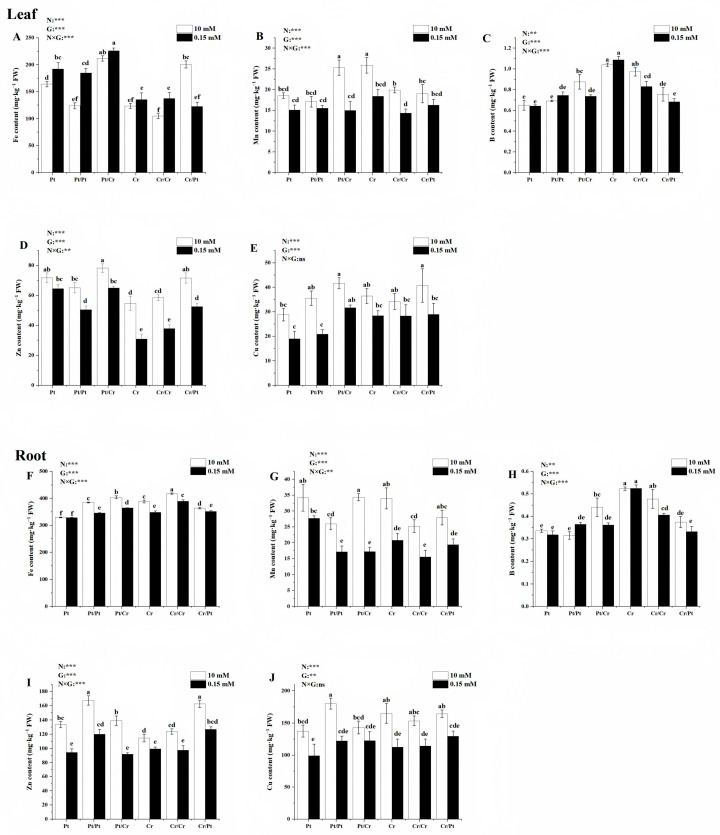
Relative levels of trace elements (Fe, Mn, B, Zn, Cu) in leaves and root of six citrus combinations under two N regimes. (**A**) Leaf Fe levels; (**B**) Leaf Mn levels; (**C**) Leaf B levels; (**D**) Leaf Zn levels; (**E**) Leaf Cu levels; (**F**) Root Fe levels; (**G**) Root Mn levels; (**H**) Root B levels; (**I**) Root Zn levels; (**J**) Root Cu levels. The six citrus combinations are: Ungrafted Trifoliate orange (*Poncitrus trifoliata* L. Raf., Pt), ungrafted red tangerine (*Citrus reticulata* Blanco, Cr), Pt grafted onto Pt (Pt/Pt), Cr grafted onto Cr (Cr/Cr), Pt grafted onto Cr (Pt/Cr), Cr grafted onto Pt (Cr/Pt). Y-axis values are instrument outputs from diluted fresh sap (YT-ZY20 tester) and are semi-quantitative relative comparisons; they were not validated by ICP-OES. Statistical significance: ** *p* < 0.01; *** *p* < 0.001; ns, not significant. Different letters indicate significant differences determined by Tukey test at *p* < 0.05. *n* = 3 biological replicates, with leaf/root tissues from two plants pooled per replicate before sap extraction.

Except for Cu, the relative levels of Fe, Mn, B and Zn in both leaves and roots were significantly affected by N treatment, graft combination, and their interaction (N × G). Under 10 mM N, relative leaf levels of Fe and Zn were significantly higher in Pt than in Cr (by 25.3% and 23.6%, respectively), whereas Mn, B and Cu levels were significantly lower in Pt than in Cr (by 28.2%, 37.5% and 21.1%, respectively). Grafted combinations generally showed higher trace element levels than self-grafted ones. For example, relative levels of Fe, Mn, B, Zn, and Cu in Pt/Cr leaves were significantly higher than in Pt/Pt leaves (by 70.9%, 47.9%, 26.5%, 20.1% and 17.1%, respectively). In Cr/Pt leaves, Fe, Zn, and Cu were higher than in Cr/Cr leaves (by 48.0%, 18.3%, and 16.1%, respectively), whereas B levels were significantly lower in Cr/Pt than in Cr/Cr (by 28.8%). Similar patterns were observed in roots (Figure 6).

Compared with 10 mM N, under 0.15 mM N, most trace element levels in leaves and roots decreased, except for a slight increase in Fe levels in Pt, Pt/Pt, Pt/Cr, Cr, and Cr/Cr leaves. Overall, under low N, no significant differences in trace element levels were observed among the six grafting combinations in either leaves or roots.

## 4. Discussion

Nitrogen is a limiting factor for the growth, development, fruit yield, and quality of citrus trees [27]. Many prior studies on different citrus rootstocks have demonstrated that they exhibit different patterns of response to soil element uptake, transport, utilization, and tolerance [34]. Roots play a vital role in responding to nitrogen availability changes and can communicate signals that lead to root modifications in plants [35]. Substantial interspecific variation exists in root system morphology and architecture among citrus species [36]. While Liu et al. [37] found no significant difference in xylem anatomy (vessel area and density) between *Citrus reticulata* Blanco var. tangerine and Trifoliate orange, the present study focused on morphological plasticity. Under low-N stress, we observed significant inhibition of plant height, stem diameter, and leaf area, with the most pronounced effects occurring in combinations using Cr rootstock (Cr/Cr and Pt/Cr). Notably, the Pt/Pt combination showed relatively small growth inhibition under low-N conditions, with total root length and root tip number increasing by 19.4% and 13.7%, respectively, compared with the Pt/Cr combination. This observation suggests that combinations with Pt rootstock may optimize root architecture (e.g., increasing root tip density and root surface area). It also suggests that when soil N availability decreases, combinations with Cr rootstock perform less well than those with Pt rootstock. Similar results were observed in ‘Xuegan’ (*Citrus sinensis* (L.) Osbeck) and ‘Shantian pummelo’ (*Citrus grandis* (L.) Osbeck) seedlings, where N deficiency inhibited plant growth, with a lesser impact on ‘Shantian pummelo’ than on ‘Xuegan’ seedlings, suggesting that ‘Shantian pummelo’ seedlings exhibit slightly higher tolerance to N deficiency [6]. Moreover, both Pt and Cr plants, when grafted onto Pt, showed higher total root length, root surface area, root tip number and root activity values compared with the plants grafted onto Cr rootstock. These results confirm that the rootstock genotype affects the horticultural characteristics of citrus [38,39]. In this study, N deficiency resulted in delayed root growth and leaf development, especially for combinations with Cr rootstock, indicating that Cr was more affected under low-N conditions, possibly in relation to its nitrogen acquisition under the conditions tested. Root morphological traits varied significantly among rootstock genotypes: Pt exhibited greater total root length, whereas Cr showed a larger average root diameter. Similar differences in average root diameter and specific root length among citrus species have been documented [36]. The root scanning data further suggest that the fine-root development characteristic of Pt rootstock (average root diameter 0.45–0.66 mm) is more conducive to forming a high specific surface area than Cr rootstock (0.48–0.70 mm), and this morphological plasticity may be associated with the observed differences in N acquisition among combinations.

Nitrogen also serves as a crucial structural component of chlorophyll, which is required for photosynthesis [6]. Many studies have indicated that N deficiency can cause chlorophyll levels to drop, leading to leaves yellowing [27], and photosynthetic capacity declines under unfavorable N supply conditions [6]. Chlorophyll plays a crucial role in converting light energy into chemical energy, impacting processes such as light absorption, transmission, and transformation necessary for photosynthesis [40]. Consistent with these observations, our research revealed a decline in photosynthetic efficiency of citrus seedlings as N concentration diminished, an effect that held true for both ungrafted and grafted plants. Notably, grafting generally induced greater chlorophyll accumulation compared with ungrafted plants, albeit with lower *P*_n_ under the corresponding conditions. The light-response curve is a critical indicator of photochemical efficiency and electron transport dynamics in plants [41]. Our research revealed the specific LSP of different grafting combinations under low-N conditions: Pt/Pt seedlings maintained stable *P*_n_ at PPFD > 1200 µmol·m^−2^·s^−1^, whereas Cr/Cr seedlings reached stable *P*_n_ at significantly lower irradiance (PPFD > 400 µmol·m^−2^·s^−1^). The heterografted combinations Pt/Cr (LSP = 1000 µmol·m^−2^·s^−1^) and Cr/Pt (LSP = 600 µmol·m^−2^·s^−1^) demonstrated intermediate phenotypes, suggesting a strong rootstock influence on photochemical performance. This result suggests that as PPFD increased, combinations with Cr rootstock had greater difficulty utilizing electrons through photochemical processes, whereas combinations with Pt rootstock showed a better ability to cope with increased light energy. This phenomenon may be related to key photosynthetic parameters under N limitation. We found that the ETR and Fv’/Fm’ of Cr/Pt seedlings were 25% and 18.7% higher, respectively, than those of Cr/Cr, suggesting that combinations with Pt rootstock had enhanced capacity to sustain photochemical activity under high PPFD. Conversely, LSPn and AQY of Cr/Cr seedlings were 25.6% and 15.8% lower, respectively, than those of Cr/Pt. This suggests that combinations with Cr rootstock may have reduced photochemical efficiency, potentially indicating greater susceptibility to photoinhibitory stress under excessive irradiance. We also found that combinations with Pt rootstock (Pt, Pt/Pt, Cr/Pt) had higher LSPn and LSP and lower LCP than combinations with Cr rootstock (Cr, Cr/Cr, Pt/Cr) under both N treatments. These variation patterns in photosynthetic characteristics may be related to inherent genetic and physiological differences [42].

Balanced nutritional management is a key factor governing fruit tree productivity and fruit quality [43]. Substantial evidence indicates that rootstock genotypes are associated with differences in foliar mineral composition, with optimal performance being rootstock–scion combination dependent [44,45]. These nutritional differences stem from variations in rootstock characteristics, including water and nutrient uptake-related traits and root system architecture [46]. Furthermore, rootstock–scion interactions modulate whole-plant physiology, affecting water relations, gas exchange, and vegetative growth. Within citrus species, substantial diversity exists in root systems morphology and architecture. As reported in previous studies, root morphological polymorphism affects nutrient accumulation in leaves, as there are inherent differences in the absorption rate, transport to leaves, and selectivity of root tissue in ion accumulation [47]. Different rootstock genotypes differ in their relative mineral element profiles. For example, Han et al. [48] pointed out that red tangerine (*Citrus reticulate* Blanco) performs better under conditions of Mg and B deficiency, whereas fragrant citrus (*Citrus junos* Sieb.ex Tanaka) is more effective under Fe deficiency. Fan et al. [49] demonstrated that Zhique (*Citrus wilsonii* Tanaka) tolerates Fe deficiency better than Trifoliate orange (*Poncirus trifoliata* L. Raf.) under calcareous soil conditions, possibly because *HA6* transcript levels are much higher in Zhique, leading to stronger H^+^ extrusion and greater rhizosphere acidification. Lu et al. [50] reported that using *Citrus reticulata* Blanco var. tangerine as an interstock can enhance the growth of ‘Newhall’ sweet orange trees grafted onto Trifoliate orange rootstocks. Sorgonà et al. [20] compared four citrus rootstocks and identified sour orange as nitrate-efficient and sweet orange as nitrate-inefficient. In the present study, N concentration had a significant effect on the relative levels of macro- and micronutrients in leaf and root tissues. Specifically, when N concentration decreased, the relative levels of P, K, Ca, and Mg in plant tissues increased, whereas N levels decreased significantly. It is worth noting that S accumulation patterns showed differential responses among grafting combinations. Consistent with our findings, previous studies have emphasized that rootstocks significantly influence macro- and micronutrient profiles in citrus [36,51,52], apple [53], and peach [44]. Differences in leaf macro- and micronutrient levels across grafting combinations may be associated with root morphological traits [54], which is in turn linked to root spread area, fine root density, root exudates, root volume, and root structure [55,56]. When examining genotype-mediated differences in nutrient profiles, we also observed organ-specific distribution patterns. For example, across both 0.15 and 10 mM N concentration, all combinations consistently exhibited 1.3-to-2.8-fold-higher N levels in leaves than in roots. Phosphorus distribution showed genotype-specific divergence: Pt and Cr/Pt combinations exhibited preferential foliar P accumulation, whereas the remaining four combinations showed significantly higher P levels in roots than in leaves under equivalent conditions. These observations suggest that the grafting combination influences the partitioning of mineral elements between shoot and root tissues.

To maintain constant K^+^ and Ca^2+^ levels in the low-N treatment, CaCl_2_ and KCl replaced Ca(NO_3_)_2_ and KNO_3_ [57]. The imposed Cl^−^ concentration in our low-N solution was 9.85 mM, which is below the toxic range (32 mM) reported for *Poncirus trifoliata* in a recent study [58]. However, chloride is biologically active and can act as a beneficial macronutrient at low concentrations rather than being an inert balancing ion. Therefore, chloride itself remains a potential confounder that was not fully separable from N limitation in this experimental design. Future studies using other anions (e.g., gluconate or sulfate) to balance K^+^ and Ca^2+^ would help disentangle these effects. Regarding mineral analysis, the YT-ZY20 tester measures available nutrients in fresh sap without digestion. It provides reliable relative comparisons (RSD < 0.2%) but not absolute ionomic values. We did not validate these measurements against ICP-OES; therefore, the reported values should be interpreted as semi-quantitative relative profiles rather than absolute tissue mineral concentrations. The conclusions drawn from these data are limited to comparative assessments among treatments and grafting combinations within this experimental system.

## 5. Conclusions

Under the controlled nutrient-solution conditions of this study, ungrafted Trifoliate orange (Pt) seedlings showed better performance under low-N stress than ungrafted red tangerine (Cr) seedlings, as evidenced primarily by the maintenance of plant height, total root length, root tip number, root activity and photosynthetic parameters. Grafting altered the responses to low-N stress, with the Cr/Cr combination showing the most pronounced limitations in root morphology, photosynthesis, pigment contents, and mineral element profiles. Among the grafted combinations, those with Pt rootstock (Pt/Pt and Cr/Pt) exhibited better performance under low-N stress than those with Cr rootstock (Cr/Cr and Pt/Cr) within this experimental system, as reflected by root morphology, photosynthetic capacity, and relative mineral element profiles. These findings provide a physiological basis for understanding grafting-combination-specific responses to low-N stress under controlled conditions.

Given the hydroponic-like pot system and the semi-quantitative nature of the mineral analysis, these results should be considered hypothesis-generating. Further validation in soil-based or field experiments is needed to assess the applicability of these findings to commercial citrus production.

## Figures and Tables

**Table 1:** Changes in morphological traits of citrus seedlings under different treatments.

N Treatment (N)	Graft Combination (G)	Height (cm)	Stem Diameter (mm)	Leaf Area (cm^2^)	SPAD
10 mM	Pt	82.7 ± 10.37 a	0.239 ± 0.024 a	6.86 ± 0.40 c	86.83 ± 1.10 a
	Pt/Pt	79.46 ± 5.15 a	0.232 ± 0.005 ab	9.85 ± 0.54 c	81.83 ± 1.60 a
	Pt/Cr	62.73 ± 3.06 b	0.267 ± 0.033 a	7.67 ± 1.15 c	83.00 ± 0.20 a
	Cr	48.26 ± 1.15 bc	0.177 ± 0.009 c	20.51 ± 2.93 a	63.56 ± 3.36 c
	Cr/Cr	37.86 ± 4.57 c	0.189 ± 0.006 bc	16.17 ± 1.16 b	58.63 ± 1.02 c
	Cr/Pt	57.16 ± 1.60 b	0.172 ± 0.003 c	24.05 ± 1.32 a	72.20 ± 2.51 b
0.15 mM	Pt	64.63 ± 4.04 a	0.183 ± 0.016 ab	5.62 ± 0.34 c	75.70 ± 5.18 a
	Pt/Pt	67.00 ± 4.07 a	0.207 ± 0.016 a	7.91 ± 0.94 c	70.53 ± 1.65 ab
	Pt/Cr	54.97 ± 5.53 ab	0.182 ± 0.040 ab	6.39 ± 0.52 c	65.53 ± 2.10 bc
	Cr	30.50 ± 7.57 cd	0.147 ± 0.011 b	15.11 ± 2.01 b	56.16 ± 3.28 d
	Cr/Cr	25.80 ± 8.37 d	0.172 ± 0.005 ab	14.83 ± 0.41 b	61.80 ± 1.04 cd
	Cr/Pt	41.90 ± 3.13 bc	0.167 ± 0.002 ab	20.46 ± 0.88 a	54.50 ± 2.47 d
N		***	***	***	***
G		***	***	***	***
N × G		ns	*	ns	**

Note: Data in the columns are the mean ± standard error (*n* = 3). Different letters indicate significant differences determined by Tukey test at *p* < 0.05. Results of a two-way ANOVA are indicated; * *p* < 0.05; ** *p* < 0.01; *** *p* < 0.001; ns, not significant, the same as below. *n* = 3 biological replicates, with the two plants per replicate averaged for growth parameters.

**Table 2:** Root morphology and root activity of citrus seedlings under different treatments.

N Treatment (N)	Graft Combination (G)	Total Root Length (cm)	Surface Area (cm^2^)	Diameter (cm)	Volume (cm^3^)	Root Tip Number	Root Activity (µg·g^−1^·h^−1^ FW)
10 mM	Pt	4035 ± 75.91 a	628 ± 18.05 a	0.66 ± 0.008 b	10.67 ± 0.12 a	33,881 ± 593 c	113.32 ± 2.16 a
	Pt/Pt	3474 ± 61.03 b	526 ± 4.57 c	0.52 ± 0.015 d	6.66 ± 0.22 d	46,948 ± 917 a	97.02 ± 2.84 b
	Pt/Cr	3061 ± 26.80 c	517 ± 10.97 c	0.59 ± 0.008 c	7.32 ± 0.14 c	39,606 ± 363 b	73.17 ± 1.99 c
	Cr	3013 ± 127.72 c	524 ± 7.90 c	0.70 ± 0.011 a	9.36 ± 0.12 b	30,428 ± 813 d	100.24 ± 2.46 b
	Cr/Cr	3069 ± 32.52 c	568 ± 18.77 b	0.54 ± 0.011 d	7.13 ± 0.13 cd	40,443 ± 564 b	79.24 ± 2.05 c
	Cr/Pt	3600 ± 66.62 b	619 ± 16.47 a	0.64 ± 0.011 b	9.48 ± 0.34 b	35,309 ± 1103 c	102.20 ± 8.62 ab
0.15 mM	Pt	3064 ± 132.09 b	554 ± 23.11 a	0.45 ± 0.007 d	6.26 ± 0.21 c	59,602 ± 983 a	86.35 ± 4.82 a
	Pt/Pt	3292 ± 22.72 a	449 ± 24.56 c	0.48 ± 0.009 bc	5.54 ± 0.09 d	51,179 ± 1097 b	85.06 ± 1.59 a
	Pt/Cr	2757 ± 66.24 c	473 ± 21.70 bc	0.48 ± 0.010 c	5.36 ± 0.10 d	45,012 ± 387 d	66.55 ± 1.68 b
	Cr	2396 ± 31.96 d	530 ± 2.70 a	0.59 ± 0.008 a	7.45 ± 0.17 a	36,946 ± 1356 e	73.45 ± 2.92 b
	Cr/Cr	2901 ± 86.81 bc	512 ± 19.97 ab	0.48 ± 0.014 bc	6.51 ± 0.16 c	50,142 ± 845 bc	67.11 ± 1.00 b
	Cr/Pt	3032 ± 49.31 b	557 ± 7.40 a	0.50 ± 0.009 b	7.05 ± 0.08 b	47,773 ± 698 c	67.42 ± 2.55 b
N		***	***	***	***	***	***
G		***	***	***	***	***	***
N × G		***	**	***	***	***	***

Note: Data in the columns are the mean ± standard error (*n* = 3). Different letters indicate significant differences determined by Tukey test at *p* < 0.05. Results of a two-way ANOVA are indicated; ** *p* < 0.01; *** *p* < 0.001. *n* = 3 biological replicates, with roots from two plants scanned separately and the values averaged per replicate.

**Table 3:** Photosynthetic parameters and chlorophyll fluorescence in citrus seedlings under different treatments.

N Treatment (N)	Graft Combination (G)	*P*_n_ (µmol·m^−2^·s^−1^)	C_i_ (µmol·mol^−1^)	Gs (mol·m^−2^·s^−1^)	ETR (µmol·m^−2^·s^−1^)	Fv/Fm	Fv’/Fm’
10 mM	Pt	11.68 ± 0.55 a	316.75 ± 6.70 a	0.168 ± 0.003 a	181.23 ± 5.51 a	0.827 ± 0.001 a	0.463 ± 0.010 a
	Pt/Pt	10.17 ± 0.84 bc	290.64 ± 1.37 b	0.141 ± 0.001 b	161.36 ± 4.54 b	0.817 ± 0.001 b	0.478 ± 0.006 a
	Pt/Cr	8.83 ± 0.27 d	274.75 ± 3.22 c	0.109 ± 0.007 d	153.70 ± 5.30 b	0.805 ± 0.001 d	0.436 ± 0.005 b
	Cr	10.69 ± 0.13 ab	257.08 ± 6.58 d	0.132 ± 0.004 bc	105.29 ± 4.56 c	0.815 ± 0.001 bc	0.359 ± 0.005 d
	Cr/Cr	8.96 ± 0.40 cd	215.29 ± 5.55 e	0.083 ± 0.001 e	94.84 ± 1.04 c	0.785 ± 0.002 e	0.352 ± 0.004 d
	Cr/Pt	8.35 ± 0.13 d	247.84 ± 4.82 d	0.127 ± 0.003 c	96.64 ± 4.92 c	0.813 ± 0.001 c	0.407 ± 0.012 c
0.15 mM	Pt	7.28 ± 0.14 a	268.97 ± 1.96 b	0.127 ± 0.003 a	120.24 ± 2.83 a	0.753 ± 0.004 ab	0.358 ± 0.014 a
	Pt/Pt	6.73 ± 0.12 ab	295.19 ± 1.65 a	0.105 ± 0.004 b	107.85 ± 1.79 b	0.749 ± 0.017 abc	0.359 ± 0.016 a
	Pt/Cr	4.99 ± 0.07 d	221.07 ± 4.46 c	0.089 ± 0.007 c	84.58 ± 2.31 c	0.719 ± 0.010 c	0.350 ± 0.027 ab
	Cr	5.35 ± 0.28 cd	209.24 ± 5.29 c	0.064 ± 0.003 d	55.30 ± 0.97 d	0.762 ± 0.005 a	0.310 ± 0.003 b
	Cr/Cr	5.02 ± 0.04 d	166.81 ± 4.30 e	0.046 ± 0.003 e	43.20 ± 2.30 e	0.731 ± 0.015 bc	0.262 ± 0.017 c
	Cr/Pt	5.97 ± 0.65 bc	190.70 ± 7.69 d	0.059 ± 0.003 d	54.00 ± 1.53 d	0.747 ± 0.010 abc	0.311 ± 0.011 b
N		***	***	***	***	***	***
G		***	***	***	***	***	***
N × G		***	***	***	***	*	***

Note: (*P*_n_-µmol·m^−2^·s^−1^) net CO_2_ assimilation, (Gs-mol·m^−2^·s^−1^) leaf stomatal conductance, (C_i_-µmol·mol^−1^) internal CO_2_, (ETR-µmol·m^−2^·s^−1^) photosynthetic electron transfer rate, (Fv/Fm) dark-adapted maximal quantum efficiency of PS II photochemistry, (Fv’/Fm’) the maximal quantum efficiency of PS II photochemistry in the light in plants of: ungrafted Trifoliate orange (*Poncitrus trifoliata* L. Raf., Pt) and red tangerine (*Citrus reticulata* Blanco, Cr), Pt grafted onto Pt (Pt/Pt), Cr grafted onto Cr (Cr/Cr), Pt grafted onto Cr (Pt/Cr), Cr grafted onto Pt (Cr/Pt). * *p* < 0.05; *** *p* < 0.001. Data in the columns are the mean ± standard error (*n* = 3). For each parameter, *n* = 3 biological replicates, with one leaf measured from one of the two plants per replicate. Different letters indicate significant differences determined by Tukey test at *p* < 0.05.

**Table 4:** Light-response parameters of leaf photosynthesis in different citrus combinations.

N Treatment (N)	Graft Combination (G)	LS*P*_n_ (µmol·m^−2^·s^−1^)	LSP (µmol·m^−2^·s^−1^)	LCP (µmol·m^−2^·s^−1^)	LSP-LCP (µmol·m^−2^·s^−1^)	AQY
10 mM	Pt	9.08 ± 0.27 a	1705.81 ± 84.38 a	21.08 ± 0.43 c	1684.73 ± 84.72 a	0.041 ± 0.001 a
	Pt/Pt	9.07 ± 0.02 a	1579.81 ± 34.75 b	16.84 ± 0.01 d	1562.97 ± 34.75 a	0.037 ± 0.001 b
	Pt/Cr	6.45 ± 0.11 c	1450.75 ± 49.52 c	35.73 ± 0.50 a	1415.03 ± 49.37 b	0.027 ± 0.001 d
	Cr	6.35 ± 0.04 c	1635.53 ± 23.00 ab	16.05 ± 0.44 d	1619.49 ± 23.00 a	0.030 ± 0.002 c
	Cr/Cr	6.16 ± 0.08 c	933.86 ± 25.86 d	24.79 ± 0.54 b	909.08 ± 26.05 c	0.023 ± 0.001 d
	Cr/Pt	8.33 ± 0.16 b	973.88 ± 5.85 d	23.82 ± 0.65 b	950.07 ± 6.38 c	0.032 ± 0.001 c
0.15 mM	Pt	7.75 ± 0.05 a	1567.13 ± 62.00 a	37.42 ± 0.35 b	1529.71 ± 111.89 a	0.030 ± 0.001 a
	Pt/Pt	6.86 ± 0.08 c	1261.61 ± 40.62 b	23.40 ± 0.02 e	1238.21 ± 40.61 b	0.024 ± 0.002 b
	Pt/Cr	4.23 ± 0.16 f	1105.38 ± 17.05 c	60.07 ± 0.68 a	1045.31 ± 16.90 c	0.017 ± 0.001 cd
	Cr	4.88 ± 0.07 e	1555.19 ± 28.61 a	35.42 ± 1.08 c	1519.78 ± 28.95 a	0.017 ± 0.001 cd
	Cr/Cr	5.41 ± 0.02 d	852.20 ± 9.99 d	34.90 ± 0.70 c	817.30 ± 9.82 d	0.016 ± 0.001 d
	Cr/Pt	7.27 ± 0.10 b	909.54 ± 6.66 d	31.62 ± 0.60 d	877.92 ± 6.59 d	0.019 ± 0.001 c
N		***	***	***	***	***
G		***	***	***	***	***
N × G		***	***	***	***	***

Note: (LSPn-µmol·m^−2^·s^−1^) maximum photosynthetic rates at the light saturation point, (LSP-µmol·m^−2^·s^−1^) the light saturation point, (LCP- µmol·m^−2^·s^−1^) light compensation point, (AQY) apparent quantum efficiency. Data in the columns are the mean ± standard error (*n* = 3). For each parameter, *n* = 3 biological replicates, with one leaf measured from one of the two plants per replicate. Different letters indicate significant differences determined by Tukey test at *p* < 0.05. Results of a two-way ANOVA are indicated; *** *p* < 0.001.

**Table 5:** CO_2_ response parameters of leaf photosynthesis in different citrus combinations.

N Treatment (N)	Graft Combination (G)	CS*P*_n_ (µmol·m^−2^·s^−1^)	Vcmax (µmol·m^−1^·s^−1^)	CSP (µmol·mol^−1^)	CCP (µmol·mol^−1^)
10 mM	Pt	13.44 ± 1.21 b	30.08 ± 1.88 a	2216 ± 31 a	65.61 ± 1.22 ab
	Pt/Pt	16.57 ± 0.77 a	35.90 ± 1.59 a	1974 ± 39 b	63.37 ± 0.78 c
	Pt/Cr	12.04 ± 0.72 bc	24.79 ± 1.61 b	1245 ± 175 c	66.28 ± 0.87 a
	Cr	12.30 ± 0.62 bc	25.31 ± 3.32 b	1185 ± 33 c	61.39 ± 0.81 c
	Cr/Cr	10.18 ± 0.91 c	28.98 ± 1.25 b	1053 ± 46 cd	65.86 ± 1.30 ab
	Cr/Pt	13.71 ± 0.16 b	17.26 ± 0.87 c	1115 ± 60 c	56.95 ± 0.22 d
0.15 mM	Pt	10.68 ± 0.46 ab	24.35 ± 0.77 a	1662 ± 50 a	81.53 ± 1.80 ab
	Pt/Pt	13.85 ± 1.80 a	24.23 ± 1.05 a	1061 ± 42 b	67.87 ± 2.04 c
	Pt/Cr	12.07 ± 1.89 ab	18.04 ± 0.77 b	1071 ± 77 b	87.67 ± 3.52 a
	Cr	11.88 ± 0.64 ab	23.18 ± 1.68 a	932 ± 40 c	78.84 ± 0.45 b
	Cr/Cr	9.74 ± 0.53 b	14.13 ± 1.49 c	963 ± 44 bc	81.51 ± 4.03 ab
	Cr/Pt	10.78 ± 0.64 ab	13.98 ± 1.08 c	903 ± 20 c	58.78 ± 1.12 d
N		***	***	***	***
G		***	***	***	***
N × G		*	***	***	***

Note: (CS*P*_n_-µmol·m^−2^·s^−1^) the maximum photosynthetic rate at CO_2_ saturation point, (Vcmax-µmol·mol^−1^) maximum carboxylation rate limited by Rubisco, (CSP-µmol·mol^−1^) CO_2_ saturation point, (CCP-µmol· mol^−1^) CO_2_ compensation point. Data in the columns are the mean ± standard error (*n* = 3). For each parameter, *n* = 3 biological replicates, with one leaf measured from one of the two plants per replicate. Different letters indicate significant differences determined by Tukey test at *p* < 0.05. Results of a two-way ANOVA are indicated; * *p* < 0.05; *** *p* < 0.001.

## Data Availability

The original contributions presented in this study are included in the article. Further inquiries can be directed to the corresponding author.
